# Reveal the Mechanisms of Yi-Fei-Jian-Pi-Tang on Covid-19 through Network Pharmacology Approach

**DOI:** 10.1155/2022/1493137

**Published:** 2022-07-16

**Authors:** Wanying Lang, Feng Yang, Fanfan Cai, Wengui Shi, Min Dong, Qi An, Yanping Li

**Affiliations:** ^1^The Department of Pain Treatment, Gansu Provincial Hospital of TCM, Lanzhou, Gansu 730050, China; ^2^Cui-Ying Biomedical Research Center, Lanzhou University Second Hospital, Lanzhou, Gansu 730030, China; ^3^The Department of Pulmonology, Gansu Provincial Hospital of TCM, Lanzhou, Gansu 730050, China

## Abstract

**Objectives:**

The Traditional Chinese Medicine (TCM) formula Yi-Fei-Jian-Pi-Tang (YFJPT) has been demonstrated effective against Corona Virus Disease 2019 (Covid-19). The aim of this article is to make a thorough inquiry about its active constituent as well as mechanisms against Covid-19 via TCM network pharmacology.

**Methods:**

All the ingredients of YFJPT are obtained from the pharmacology database of the TCM system. The genes which are associated with the targets are obtained by utilizing UniProt. The herb-target network is built up by utilizing Cytoscape. The target protein-protein interaction network is built by utilizing the STRING database and Cytoscape. The critical targets of YFJPT are explored by Gene Ontology (GO) and the Kyoto Encyclopedia of Genes and Genomes (KEGG).

**Results:**

The outcomes show that YFJPT might has 33 therapeutic targets on Covid-19, namely, interleukin 2 (IL2), heme oxygenase 1 (HMOX1), interleukin 4 (IL4), interferon gamma (FNG), *α* nuclear factor of kappa light polypeptide gene enhancer in Bcells inhibitor, alpha (NFKBIA), nuclear factor-k-gene binding (NFKB), nitric oxide synthase 3 (NOS3), intercellular adhesion molecule 1 (ICAM1), hypoxia inducible factor 1 subunit alpha (HIF1A), mitogen-activated protein kinase 3 (MAPK3), epidermal growth factor receptor (EGFR), interleukin 10 (IL10), jun proto-oncogene (JUN), C-C motif chemokine ligand 2 (CCL2), C-X-C motif chemokine ligand 8 (CXCL8), tumor protein p53 (TP53), interleukin 1 beta (IL1B), AKT serine/threonine kinase 1 (AKT1), tumor necrosis factor (TNF), interleukin 6 (IL6), erb-b2 receptor tyrosine kinase 2 (ERBB2), RELA proto-oncogene (RELA), NF-*κ*B subunit, caspase 8 (CASP8), peroxisome proliferator activated receptor alpha (PPARA), TIMP metallopeptidase inhibitor 1 (TIMP1), transforming growth factor beta 1 (TGFB1), interleukin 1 alpha (IL1A), signal transducer and activator of transcription 1 (STAT1), mitogen-activated protein kinase 8 (MAPK8), myeloperoxidase (MPO), matrix metallopeptidase 3 (MMP3), matrix metallopeptidase 1 (MMP1), and NFE2 like bZIP transcription factor 2 (NFE2L2). The gene enrichment analysis prompts that YFJPT most likely contributes to patients related to Covid-19 by regulating the pathways of cancers.

**Conclusions:**

That will lay a foundation for the clinical rational application and further experimental research of YFJPT.

## 1. Introduction

The infection of a new beta-coronavirus which is the severe acute respiratory syndrome coronavirus-2 (SARS-CoV-2) and affecting the socioeconomic conditions of billions of people worldwide spreads around the world and results in a potentially lethal disorder: the Corona Virus Disease 2019 (Covid-19) since December 2019 [1–3]. A large quantity of patients who are influenced suffer from pneumonia (which is named novel coronavirus pneumonia, NCP) and soon develop a severe acute respiratory insufficiency which is likely to result in a bad prognosis as well as an excess death rate [4–6]. More than 2.7 billion infections and 5,336,647 deaths have been confirmed nationwide, which has made the United States the hardest-hit country in the world by the end of 2021 [7–9]. Compared to the patients who suffer from SARS-CoV or MERS-CoV, Covid-19 which has a higher mortality is far more transmissible because the secondary case produced by each case that the infected is 2.68 on average. Governments all around the world have carried out a series of measures with the intention of suppressing the spread of the disease [10]. To date, no specific antiviral treatment is available for Covid-19. In recent years, the values of TCM are more and more appreciated [11–13]. YFJPT is recommended for the last stages of the disease in the latest clinical guideline for Covid-19. YFJPT composes of 10 herbs [14–17]. The herbs and dosages are as follows: Radix Bupleuri 15 g, Dried Tangerine 10 g, Platycodon Grandiflorum 10 g, Amomum Villosum 10 g, Malt 15 g, Radix Astragali 30 g, Codonopsis Pilosula 15 g, Radix Angelicae Sinensis 10 g, Actractylodes Macrocephala Koidz 15 g, and Radix Paeoniae Alba 10 g. However, the specific mechanism is still unclear [18, 19]. As one of the new approaches for the discovery of novel drug on the foundation of single-target drug study recently, network pharmacology could figure out the issue [20–23]. As a result, the combination of the emergent network science and TCM can offer new approaches and opportunities in terms of discovering biomarkers and bioactive ingredients, disclosing the potential action mechanisms as well as probing the empirical evidence of herbal formulae based on the complicated biological systems [24–27]. However, this is still one of the threats for evidence-based TCM understanding the scientific foundation of TCM herbal formulae at the molecular level and from a systemic perspective. It could classify the active constituents in TCMs as well as their biological targets by utilizing leading-edge technologies which are applied in analytical chemistry and chemical biology with the intention of illustrating commonly utilized herbs or herbal formulae. It is necessary to have a better comprehension of by which way various ingredients in the herbal formula work together and what influence they will have on various targets of a disease because it is important in the biological and clinical level for the investigation of TCM in modern day, although according to some researches, a single active constituent is successfully extracted from an herb or herbal formula, and its biological activities and targets have been identified [28–30]. It is possible for these phenotypic data to be analyzed in the structure of the network of molecular interaction, so as to reveal the associations of diseases as well as the disease phenotypes which have not been recognized as well as choosing the pharmacotherapeutics or classifying the underlying protein-drug. In this paper, we will investigate the molecular foundation of herbal formulae, which will contribute to the increase of the acceptance of TCM all over the world. These attempts have promoted the recognition of the major active constituents as well as the synergistic constituent pairs and, under some cases, have resulted in drug discoveries on the foundation of TCM [31–33].

## 2. Methods

### 2.1. Chemical Compounds in YFJPT

We use the Traditional Chinese Medicine Integrated Database (TCMID, https://www.megabionet.org/tcmid/), which is the Traditional Chinese Medicine integrative database for herb molecular mechanism analysis, to achieve the aim of collecting the compounds of YFJPT; the Traditional Chinese Medicine Systems Pharmacology Database (TCMSP, https://lsp.nwu.edu.cn/), which is a typical system pharmacology platform that is designed for Chinese herbal medicines; and the TCM Database@Taiwan (https://tcm.cmu.edu.tw/) as the most comprehensive TCM database universally [34]. Eventually, after deleting the duplicate data, we retrieve 104 herbal compounds.

### 2.2. Targets for YFJPT

**Figure 1:**
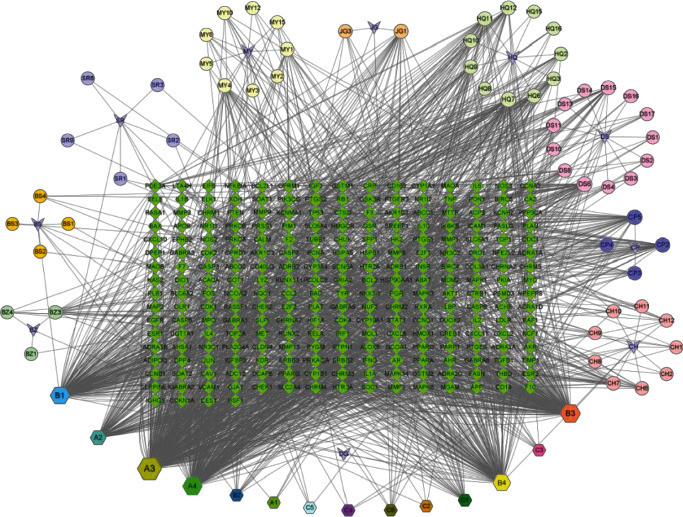
Chinese herbal medicinal ingredient prescription (YFJPT)-target network.

UniProt (https://www.uniprot.org) provides information on chemical substances and biological activities. We make a decision to eliminate some chemicals after the replicated data is deleted due to the lack of accurate structural information of the targets of the compounds, which is unable to be forecasted successfully [35, 36] (Figure 1).

### 2.3. Covid-19 Targets

**Figure 2:**
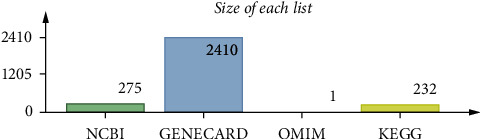
Covid-19 targets of different databases.

The genes in relation to Covid-19 which are different are obtained from National Center for Biotechnology Information (NCBI) (https://www.ncbi.nlm.nih.gov), Online Mendelian Inheritance in Man(OMIM) (https://www.omim.org), GeneCards (https://www.genecards.org), and Kyoto Encyclopedia of Genes and Genomes(KEGG) (https://www.genome.jp/kegg) [37, 38] (Figure 2).

### 2.4. Protein-Protein Interaction Data

**Figure 3:**
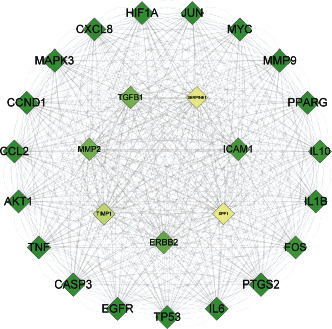
Yi-Fei-Jian-Pi-Tang (YFJPT) targets PPI network.

The STRING (https://string-db.org) with the species which are confined to “Homo sapiens” extracts the data of protein-protein interaction (PPI). Meanwhile, STRING database defined PPI with confidence ranges for data scores (high: >0.7; medium: 0.4–0.7; low confidence: scores <0.4), which is a database of predicted protein-protein interplay [39–42]. PPIs with composite scores >0.7 are extracted in this essay on the basis of these scores (Figure 3).

### 2.5. Network Construction

**Figure 4:**
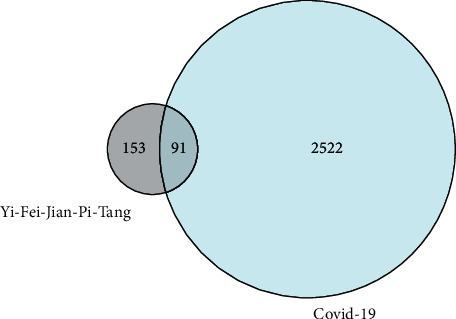
Common genes between YFJPT and Covid-19 venny.

**Figure 5:**
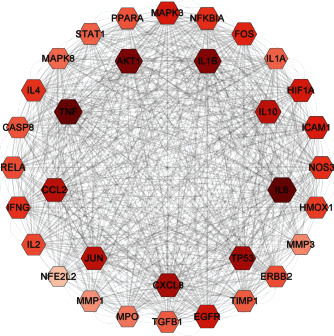
Chinese herbal medicinal ingredient prescription (YFJPT) target Covid-19 PPI network.

**Figure 6:**
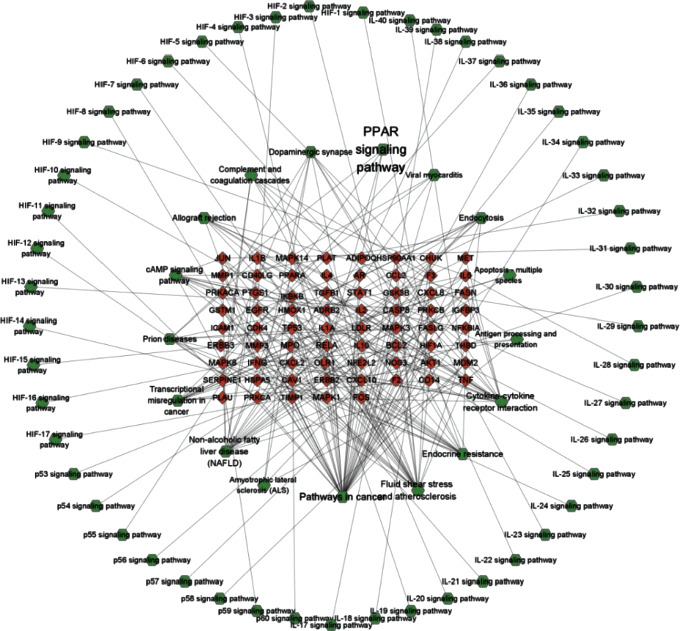
Pathway-target network.

The network visualization software Cytoscape (https://cytoscape.org/, ver. 3.9.0) could fit the visualizing networks of intermolecular interactions well, so it is utilized with the intention of displaying all of the networks presented below and provided a powerful suit of visualization functions, data analyzing and integration to make an analysis for the complex networks [6, 43–45] (Figures 4–6).

### 2.6. Gene Ontology and Pathway Enrichment

**Figure 7:**
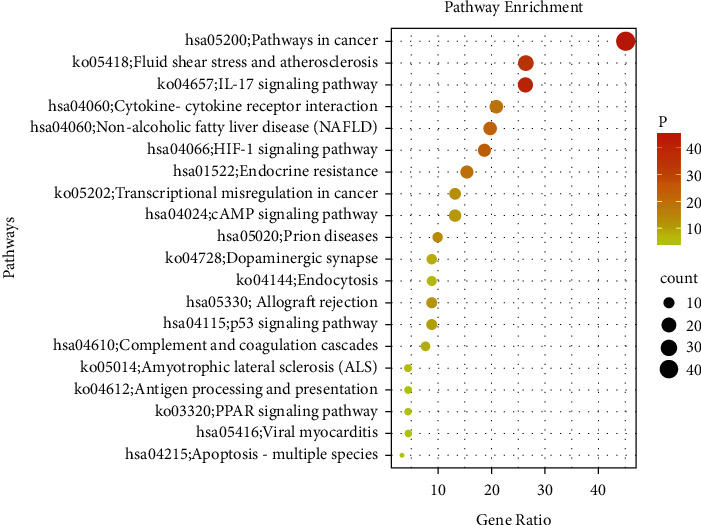
KEGG analysis for the major targets of YFJPT.

**Figure 8:**
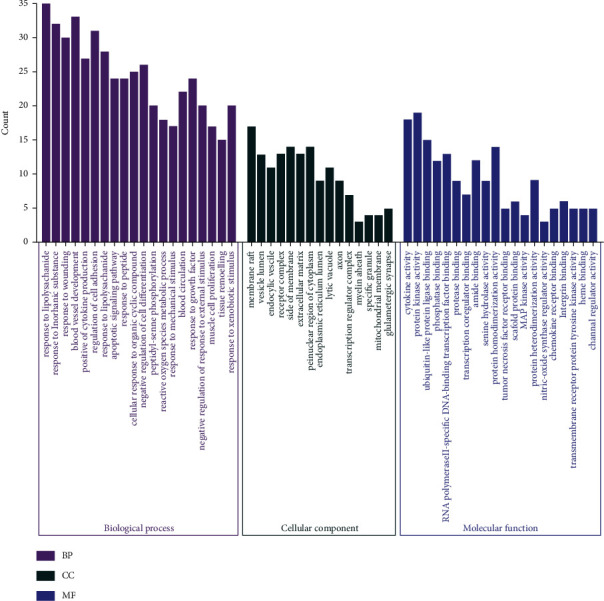
GO (BP, CC, MF) analysis for the major targets of YFJPT.

The Database for Metascape (https://metascape.org/gp/index.html) is applied to achieve the aim of performing KEGG pathway enrichment analysis and GO, which provides a biologist-oriented resource for the investigation of systems-level datasets. Enriched GO terms and pathways are dealed by utilizing the WeiShengXin tools (https://www.bioinformatics.com.cn) which is an online platform free of cost for database analyzing [46–49] (Figures 7 and 8).

It can be seen from the *y*-axis that the KEGG paths of the target genes are obviously enriched, and the *x*-axis reveals the gene ratio. Gene ratio refers to the ratio of the quantity of target genes which belongs to a pathway to the quantity of all the interpreted genes which are located in the path. The higher gene ratio stands for the higher concentration level. The size of the dot indicates the quantity of target genes in the path, and the color of the dot presents the different *p* value.

The *x*-axis presents the GO sorts of the significantly enriched target genes, and the *y*-axis presents quantities of the target genes.

## 3. Results

### 3.1. Chinese Herbal Medicinal Ingredient Prescription (YFJPT)-Target Network

The Chinese herbal medicinal ingredient prescription (YFJPT)-target network is described in Figure 1 including 328 nodes and 1705 edges. We found out 14 common ingredients in YFJPT [50–52]. A1 is the common ingredient between Radix Paeoniae Alba and Radix Astragali. A2 and A3 are the common ingredients between Radix Bupleuri and Radix Astragali. A4 is the common ingredient of Platycodon Grandiflorum, Malt, and Codonopsis Pilosula. B1 is the common ingredient of Radix Paeoniae Alba, Amomum Villosum, Malt, and Radix Angelicae Sinensis. B2 is the common ingredient of Radix Paeoniae Alba, Malt, and Dried Tangerine. B3 is the common ingredient of Radix Paeoniae Alba, Radix Bupleuri, Radix Astragali. B4 is the common ingredient of Radix Bupleuri, Amomum Villosum, Radix Angelicae Sinensis, and Codonopsis Pilosula. C1 is the common ingredient between Radix Paeoniae Alba and Malt. C2 is the common ingredient between Actractylodes Macrocephala Koidz and Radix Astragali. C3 is the common ingredient between Malt and Radix Astragali. C4 is the common ingredient between Malt and Amomum Villosum. C5 is the common ingredient between Amomum Villosum and Codonopsis Pilosula. C6 is the common ingredient between Codonopsis Pilosula and Platycodon Grandiflorum. These results hint that the common genes of YFJPT which revealed the features of a multitarget, multicomponent as well as multidisease of the herbal medicine actually might influence such targets synergistically, as a result, they can contribute to the treatment of Covid-19 as well as the pulmonary system diseases.

### 3.2. Chinese Herbal Medicinal Ingredient Prescription (YFJPT) Targets' PPI Network Analysis

The YFJPT targets' PPI network is displayed in Figure 3 with 241 nodes and 9072 edges. Three topological properties of each node are determined with the intention of finding the core nodes. At last, there are 26 nodes whose average value is more than 52 chosen as core nodes, which means, ERBB2, TP53, IL6, PTGS2, FOS, IL1B, IL10, SPP1, PPARG, MMP9, MYC, JUN, HIF1A, CXCL8, ICAM1, MAPK3, CCL2, CCND1, AKT1, TNF, SERPINE1, TGFB1, MMP2, TIMP1, CASP3, and EGFR. Such genes may occupy the critical or central position in the progress of Covid-19 [53–56].

### 3.3. YFJPT Target Covid-19 PPI Network

A compound-target-Covid-19 PPI network is built up by using 33 nodes and 946 edges with the intention of analyzing the significance of compound targets after filtrating the database by the Cytoscape. It shows us an intuitive concept to distinct those core nodes with high connectivity from the others in the network [57–60]. The findings of the network investigation show that there are 33 nodes which have a value of degree ≥30, node between ≥0.002, and combined-score ≥0.4 might be regarded as main nodes, namely, IL2, HMOX1, IL4, IFNG, NFKBIA, NOS3, FOS, ICAM1, HIF1A, MAPK3, EGFR, IL-10, JUN, CCL2, CXCL8, TP53, IL-1B, AKT1, TNF, IL-6, ERBB2, RELA, CASP8, PPARA, TIMP1, TGFB1, IL1A, STAT1, MAPK8, MPO, MMP3, MMP1, and NFE2L2. As we know, YFJPT probably exerts its therapeutic effect on Covid-19 through regulating and binding certain protein targets. Except for pathways in cancer, PPAR signaling, amyotrophic lateral sclerosis, fluid shear stress and atherosclerosis, viral myocarditis, transcriptional misregulation in cancer, prion diseases, endocytosis, endocrine resistance, dopaminergic synapse, cytokine-cytokine receptor interaction, complement and coagulation cascades, apoptosis-multiple species, antigen processing and presentation, allograft rejection, and other signal pathways only have one gene.

### 3.4. GO and KEGG Enrichment Analyses

We carry out a GO enrichment analysis for the function of molecules, cellular ingredient as well as the biological process of the 30 targets which are chosen to clarify the multiple mechanisms of YFJPT on Covid-19.  Figure 7 lists the top 20 obviously enriched GO terms of such targets. The findings reveal that the targets of YFJPT are significantly relevant to 3 biological processes: response to ii-polysaccharide, blood vessel development, and regulation of cell adhesion. 3 molecular functions: cytokine activity, protein kinase activity as well as ubiquitinlike protein ligase binding. 1 cellular component: membrane raft, which proved that YFJPT is likely to act by involving the biological courses, molecular functions, and cellular ingredient. In addition, some pathways like pathways in cancer (hsa05200), IL-17 signaling pathway (ko04657), and fluid shear stress and atherosclerosis(ko05418) have been demonstrated as precise target paths for treating Covid-19. Among 20 signaling pathways, pathways in cancer (hsa05200) which is the most significant one regulates the procedures of death, multiplication, survival, and hereditary steadiness for lung cells.

## 4. Conclusion

We predict 33 potential targets in our present study, which suggests that YFJPT is a complex prescription consisting of multiple components so as to affect an enormous variety of different targets. The data investigation reveals that YFJPT is likely to apply the pharmacological influences on Covid-19 by regulating certain targets which consists of MPO, CCL2, IL2, IFNG, IL4, TP53, and NOS3. The GO investigation of targets uncovers that it is possible for the constituent of YFJPT to bring about synergistic influences for treating Covid-19 primarily through accommodating relevant biological courses, such as paths in cancer, HIF-1 signal path, IL-17 signal path, and antigen processing and presentation [61]. At the same time, the pathway analysis shows that YFJPT might work on various signal paths which are associated with the pathogenes of Covid-19 including pathways in cancer(hsa05200), IL-17 signaling pathway(ko04657), fluid shear stress and atherosclerosis(ko05418), HIF-1 signaling pathway(hsa04066), nonalcoholic fatty liver disease (hsa04932), and so on. We show that YFJPT significantly impacts various target genes transformed in patients who suffer from Covid-19 for the first time in this research, which contains the latest trends that Covid-19 is able to be applied to the gradual accumulation of various epigenetic modifications in tumor cells. On the foundation of the system analysis for the crucial targets, bioactivators, and critical paths of YFJPT against Covid-19, our research simultaneously uncovers that the features of YFJPT are phytotherapy of multicomponents and multitarget cotherapy impacts [62–64]. Furthermore, we hope that our research can be contributed to promoting new exploration of other Chinese herbs against pulmonary system diseases as well as the utilization of network pharmacology in terms of drug discovery.

## Data Availability

The data used to support the findings of this study are included within the article.
